# The neuropsychological functions of schoolchildren after the reopening of brazilian schools during the Covid-19 pandemic

**DOI:** 10.1590/2317-1782/20232022334en

**Published:** 2023-11-10

**Authors:** Maria Rebeca de Carvalho Porto Ribeiro, Letícia Corrêa Celeste, Vanessa de Oliveira Martins Reis

**Affiliations:** 1 Programa de Pós-graduação em Ciências da Reabilitação - PPGCR, Faculdade de Ceilândia - FCE, Universidade de Brasília - UnB - Brasília (DF), Brasil.; 2 Faculdade de Ceilândia - FCE, Universidade de Brasília - UnB - Brasília (DF), Brasil.

**Keywords:** Social Isolation, Flexibility, Return to School, Children, Elementary School, Cognition, Family Environment

## Abstract

**Purpose:**

The objective of this study was analyzed the neuropsychological functions of students from a public school in Brazil, enrolled in the 1st and 2nd year of Elementary School at the time of the reopening of schools during the COVID-19 pandemic and to access the influence of family and contextual information on the performance of these skills.

**Methods:**

117 students participated in the study, as well as their parents or guardians. The children were evaluated in person using the Brief Child Neuropsychological Assessment Instrument (NEUPSILIN-Inf). The parents/guardians answered remotely the Inventory of Resources of the Family Environment and questions about socioeconomic classification and maternal education.

**Results:**

The data showed a high prevalence of children who had problems or deficits in the functions of orientation, memory, language, visuospatial skills, arithmetic skills and verbal fluency. Furthermore, predictable activities that signal some degree of stability in family life are predictors of children's performance in orientation skills and resources that promote proximal processes significantly reflect on language performance. The results suggest that children included in families with a household income below one Brazilian minimum monthly salary presented poorer inhibitory control performances.

**Conclusion:**

The impact of changes in neuropsychological skills in children's learning were presented and discussed, highlighting the need for immediate and targeted intervention of these functions. Contextual factors that influenced the performance of neuropsychological skills were also considered.

## INTRODUCTION

The advent of the COVID-19 pandemic resulted in a worldwide challenge. In March 2020, the World Health Organization (WHO) declared a global pandemic, suggesting that immediate measures should be taken in order to mitigate the transmission of the virus. In this context, the governments of several countries used quarantine and social distancing as strategies to combat COVID-19^(1)^. In Brazil, the administrative autonomies of municipalities and states allowed these actions to be implemented and as a consequence schools, establishments and companies were closed for a long period bringing about changes to society and the peoples´ lives^(1)^.

Previous studies carried out after pandemics, epidemics and natural disasters have shown that there are consequences on the economy, health and in different spheres of society during the event which impacts subsequent generations^(2)^. However, educational losses, impacts on learning and the home environment following these crises, as well as the long-term consequences, have yet to be widely tracked^(2)^.

Some studies reflect on the negative effects of the COVID-19 pandemic and isolation measures in terms of teaching and learning, and researchers and organizations have projected that school closures have had a detrimental effect on children^(2-5)^, with reduction in reading fluency^(5)^ and deceleration of learning and academic gains^(3)^.

The first years of elementary school are essential for children's literacy. At this moment, it is sought that the student understands what he reads and writes and develops the necessary skills to continue this process autonomously^(6)^. Learning is impacted by the integrity of neuropsychological skills, which involve cognitive, attentional, linguistic, and memory aspects^(7)^.

Recent studies have demonstrated a relationship between reading and writing performance and linguistic-cognitive aspects that suggest that children with better performances in language and cognition tend to have better reading and writing rates^(8-10)^. A research^(9)^ conducted in 2018 with Brazilian schoolchildren showed that children with learning disabilities have deficits in memory and metacognitive skills. Another study published in 2020^(10)^, also with Brazilian students, showed that some cognitive variables, such as phonological awareness and automatic naming, are predictors of reading.

Being the individual influenced by the environment in which he is inserted, one can imagine the repercussions of the pandemic scenario and the closure of schools for the acquisition of neuropsychological skills, fundamental for the process of literacy and for good school performance.

Given the concerns of the academic community about the possible negative impacts of isolation on children and the gradual resumption of face-to-face classes, the objective of the present study was to verify and analyze the prevalence of children at the beginning of literacy, with deficits in neuropsychological functions at the time the reopening of public schools during the pandemic, as well as the influence of family and contextual factors.

## METHODS

The study included 117 children of both sexes who took part in the study, 59 from the 1st year and 58 from the 2nd year of a public elementary school in the Federal District (Brazil), as well as their parents/guardians. It was considered as inclusion criteria to be duly enrolled in the school in which the study was developed, in a regular or inclusive class. The exclusion criterion was children with uncorrected visual difficulties and intellectual and/or hearing impairments, reported by parents or guardians or by the school, and the incomplete response of the protocols.

In order to achieve the objectives of this study, parents or guardians responded remotely to an interview containing questions of general data and maternal schooling, in addition to the Family Environment Resources Inventory (RAF) and the Brazilian Economic Classification (CCEB) criterion. Data collection was performed in person through the Child Brief Neuropsychological Assessment Instrument (NEUPSILIN-Inf).

The Family Environment Resource Inventory (RAF) is composed of 10 open-item multiple-choice questions that aim to assess a child's routine and family environment^(11)^. The questionnaire is grouped into three major domains: resources that promote proximal processes, predictable activities that signal some degree of stability in family life, and parenting practices that promote family-school bonding^(11)^.

The Brazilian Economic Classification Criterion (CCEB) aims to assess the purchasing power of consumer groups, dividing them into economic classes. It consists of questions that result in a final score that classifies the respondent in classes A, B1, B2, C1, C2 and D-E^(12)^.

Children's Brief Neuropsychological Assessment Instrument (NEUPSILIN- Inf) is a short-term protocol designed to assess eight neuropsychological functions in children aged between six and 12 years. It consists of 26 subtests that involve orientation skills; attention; visual and emotion perception; verbal and visual memory, subdivided into working memory, episodic and semantic; arithmetic skills; language, composed of oral language, reading and writing; visuoconstructive skills; and executive functions, which include inhibitory control and verbal fluency^(13)^.

Participants were recruited by telephone and during the call, parents/guardians received information about the research and any doubts or doubts were clarified. For those who agreed to participate, the application of the interview was scheduled, with questions of general data and maternal schooling, and questionnaires (CCEB and RAF).

After the interview, a schedule was proposed so the child could be evaluated with NEUPSILIN-Inf at the school. With the relaxation of school closure measures after 15 months of social isolation and the creation of a well-established safety protocol, it was possible to start face-to-face collection with children in June/2021. The protocol was used following standardized commands and stimuli as stated in the application book.

The present study was approved by the Research Ethics Committee number. 2,499,005. All participants were aware of the procedures and objectives of the study and signed the free and informed consent form for parents or guardians and students and the free and informed consent form for students.

For data analysis, the NEUPSILIN-Inf sub-items received raw scores and Z scores according to the test criteria^(13)^. Each Z-score was classified as suggestive of neuropsychological deficit alertness (z-score between -1.0 and -1.5), suggestive of deficit (z score less than or equal to -1.5), suggestive of alert for moderate to severe deficit (z score between -1.6 and -2.0), and suggestive of significant deficit severity (z score less than or equal to -2.0).

In view of the absence of normative data for the population of the Federal District for the analysis of NEUPSILIN-Inf, the reference data of students from Rio Grande do Sul were considered, taking into account age (six to eight years) and type of school (public). This choice took into account the Basic Education Development Index (IDEB) for the initial years of 2019^(14)^. The Federal District presented a score of 6.1 in the IDEB for the initial years; Rio Grande do Sul 5.8; and São Paulo 6.5.

The contextual independent variables considered for the study were, maternal schooling, family environment and socioeconomic classification. Regarding maternal schooling, we considered the grouping of partial or complete completion of elementary school, high school and higher/graduate education.

For the analysis of the family environment we used the scores obtained in the three major domains of the RAF tool, grouping the questions according to the division proposed by the protocol, namely resources that promote proximal processes (questions 1,2,3,4,5,6,7), predictable activities that signal some degree of stability in family life (questions 9, 10) and parenting practices that promote the family-school bond (questions 8). Finally, for the analysis considering the socioeconomic classifications, we grouped the B1, C1 and C2 classes, which had a forecast of a household income above one minimum monthly wage, and the grouping of classes D/E, which had a household income forecast below one minimum wage.

SPSS 25.0 software was used for data analysis, considering a significance level of 5% for inferential analyses. In the descriptive analysis of the quantitative variables, measures of central tendency, variability and position were calculated.

The correlation between non-normal quantitative variables was carried out using the Spearman Correlation Test. The inferential analysis comparing non-normal quantitative variables between two independent groups was performed using the Mann-Whitney test. The inferential analysis comparing non-normal quantitative variables as a function of multiple independent groups was performed using the Kruskal-Wallis test.

Multiple linear regression analyses were performed by the backward method to predict the dependent variables raw score of performance of the neuropsychological functions of orientation, attention, perception, memory, visuospatial skills, arithmetic skills, verbal fluency, language and inhibitory control from the independent variables mother's schooling, economic criteria of Brazil, resources that promote proximal processes, family-school relationship and activities that signal some degree of family stability. Variables with multiple categories were transformed into binaries by the dummy method.

## RESULTS

The study included 117 children in the literacy phase, aged between six and eight years, with an average of six years and nine months. The students had a balanced frequency of schooling in the first (n=59; 50.43%) and second (n=58; 49.57%) years of elementary school.

Regarding the economic classification of the families, the most frequent category was C2 (n=60; 51.28%), followed by C1 (n=27; 23.08%), DE (n=26; 22.22%) and B2 (n=4; 3.42%). Our sample did not present participants classified as A and B1.

Maternal schooling was predominantly high school (n=63; 53.80%), with mothers who had Elementary School (n=12; 10.70%) and Higher Education or Graduate Studies (n=37; 33.00%).

Table 1 shows the prevalence of alertness and deficit in each of the neuropsychological functions assessed by NEUPSILIN-Inf. There were a greater number of students with a classification suggestive of alertness or neuropsychological deficit for the Z-scores of orientation, memory, language, visuospatial skills, arithmetic and verbal fluency.

**Table 1:** Prevalence of alertness and deficit in neuropsychological functions in children of the 1st and 2nd years of a public school in the Federal District -Brazil from the NEUPSILIN-Inf

**Variable and categories**	**n**	**Categories (%)**	**Total (%)**
**Guidance**			
Alert for Neuropsychological deficit	54	46.15	58.12
Major severity deficit	14	11.97	
**Attention**			
Alert for Neuropsychological deficit	13	11.11	41.88
Neuropsychological deficit	5	4.27	
Moderate to severe deficit	6	5.13	
Major severity deficit	25	21.37	
**Perception**			
Moderate to severe deficit	12	10.26	17.10
Major severity deficit	8	6.84	
**Memory**			
Alert for neuropsychological deficit	16	13.68	62.40
Neuropsychological deficit	5	4.27	
Moderate to severe deficit	10	8.55	
Major severity deficit	42	35.9	
**Language**			
Alert for neuropsychological deficit	14	11.97	62.24
Neuropsychological deficit	6	5.13	
Moderate to severe deficit	15	12.82	
Major severity deficit	46	39.32	
**Visuospatial skills**			
Alert for neuropsychological deficit	8	6.84	66.67
Moderate to severe deficit	11	9.4	
Major severity deficit	59	50.43	
**Arithmetic**			
Alert for neuropsychological deficit	39	33.33	69.23
Neuropsychological deficit	5	4.27	
Moderate to severe deficit	27	23.08	
Major severity deficit	10	8.55	
**Verbal Fluency**			
Alert for neuropsychological deficit	18	15.38	51.28
Neuropsychological deficit	2	1.71	
Moderate to severe deficit	11	9.4	
Major severity deficit	29	24.79	
**Inhibitory Control**			
Alert for neuropsychological deficit	7	5.98	29.91
Neuropsychological deficit	2	1.71	
Moderate to severe deficit	3	2.56	
Major severity deficit	23	19.66	

**Caption:** n = absolute frequency; % = relative frequency

Tables 2 and 3 show the results of the association analysis between the RAF domains and performance in neuropsychological functions. There was no correlation between gross performance scores in neuropsychological functions and the resources that promote proximal processes, parenting practices that promote family-school bonding and predictable activities that signal some degree of stability in family in the studied sample (Table 3).

**Table 2:** Descriptive analysis of the variable resources that promote proximal processes, family-school relationship, predictable activities that signal some degree of stability in family life in children and neuropsychological skills

**Variable**	**Average**	**SD**	**Minimum**	**Maximum**	**1Q**	**Median**	**3Q**
**RAF**							
Resources that promote proximal processes	29.23	10.87	5	54	20	31	38
Family-school relationship	12.24	2.46	6	18	11.5	12	13
Predictable activities that signal some degree of stability in family life	20.4	5.53	1	28	17.5	21	24
**NEUPSILIN-Inf**							
Guidance	3.21	1.06	0	6	3	3	4
Attention	44.06	7.98	19	59	38.5	45	51
Perception	5.82	0.41	4	6	6	6	6
Memory	40.59	17.12	5	85	28.5	40	51.5
Language	35.21	17.08	0	65	20	31	52
Visuospatial skills	15.58	4.06	0	23	14	16	18
Arithmetic	3.56	3.44	0	18	1	2	6
Verbal fluency	10.84	5.46	0	26	7	10	14
Inhibitory control	40.73	16.94	0	59	39	46	52

**Caption:** SD = standard deviation; 1Q = first quartile; 3Q = third quartile

**Table 3:** Correlation between the variables gross performance score in neuropsychological functions and the RAF domains

	**Resources that promote proximal processes**	
	r	**p-value**
Guidance	0.075	0.422
Attention	0.015	0.877
Perception	-0.005	0.954
Memory	0.116	0.213
Language	0.174	0.061
Visuospatial skills	-0.036	0.704
Arithmetic	0.151	0.103
Verbal fluency	-0.075	0.422
Inhibitory control	0.135	0.146
	**Family-school relationship**	
	**r**	**p-value**
Guidance	-0.080	0.392
Attention	-0.072	0.438
Perception	-0.119	0.200
Memory	-0.179	0.053
Language	-0.030	0.752
Visuospatial skills	-0.135	0.147
Arithmetic	-0.099	0.288
Verbal fluency	-0.057	0.541
Inhibitory control	-0.170	0.067
	Predictable activities that signal some degree of stability in family life	
	**r**	**p-value**
Guidance	0.156	0.094
Attention	0.052	0.574
Perception	0.050	0.594
Memory	0.081	0.386
Language	0.153	0.101
Visuospatial skills	0.052	0.580
Arithmetic	0.099	0.289
Verbal fluency	0.101	0.281
Inhibitory control	0.105	0.261

Spearman's Correlation Test.

**Caption:** r= correlation coeficiente; p-value = test of significance

In the socioeconomic analysis, children from classes B2/C1/C2 had significantly higher scores in the neuropsychological function of inhibitory control than children from classes D/E (p=0.020) (Table 4). No difference was observed in the comparison of the raw scores of performance of neuropsychological skills as a function of the education of the children's mothers.

**Table 4:** Inferential analysis of comparison of variables gross performance scores in neuropsychological functions as a function of variables of socioeconomic level

**Neuropsychological functions**	**Socioeconomic Level**	**Average**	**SD**	**Minimum**	**Maximum**	**1Q**	**Median**	**3Q**	**p-value**
Guidance	B2/C1/C2	3.18	0.98	0	6	3	3	4	0.611
	DE	3.31	1.32	1	6	2	3	4	
Attention	B2/C1/C2	43.67	7.96	19	59	38	45	50	0.372
	DE	45.42	8.09	28	59	38.75	46	51.25	
Perception	B2/C1/C2	5.82	0.41	4	6	6	6	6	0.763
	DE	5.81	0.4	5	6	6	6	6	
Memory	B2/C1/C2	41.18	15.59	5	85	30	42	51	0.364
	DE	38.54	21.89	11	74	20.5	31	63.25	
Language	B2/C1/C2	35.23	16.42	0	65	21	31	50	0.665
	DE	35.15	19.56	10	64	17	30.5	55.25	
Visuospatial skills	B2/C1/C2	15.68	3.86	0	23	14	16	18	0.775
	DE	15.23	4.77	0	22	12	15.5	18.25	
Arithmetic	B2/C1/C2	3.48	3.26	0	18	1	2	5.5	0.678
	DE	3.85	4.07	0	11	1	1	9	
Verbal fluency	B2/C1/C2	10.71	5.34	0	25	7	10	14	0.753
	DE	11.27	5.97	0	26	6	10.5	15.25	
Inhibitory control	B2/C1/C2	42.15	16.13	0	59	41	47	52	0.02*
	DE	35.73	19.02	0	53	31.25	43.5	49.25	
							

Kruskal-Wallis test

* = Statistically significant value (p < 0.05)

**Caption:** SD = standard deviation; 1Q = first quartile; 3Q = third quartile; p-value = test of significance

Multiple linear regression was carried out to verify whether the independent variables were able to predict the dependent variables gross score of orientation and gross score of language (Table 5). The analysis resulted in a statistically significant model, both for orientation *[F(1, 110) = 4.069; p=0.046; R2 = 0.036],* and for language *[F(1, 110) = 4.859; p=0.030; R2 = 0.042]. The independent variable* predictable activities that signal some degree of stability in family *(β = 0.189; t = 2.017; p = 0.046)* is a predictor of the dependent variable gross score of orientation. The equation that describes this relationship is (guidance raw score) = 2.497 + 0.036 (activities that signal some degree of stability in family)*.* The independent variable features that promote proximal processes *(β = 0.206; t = 2.204; p = 0.030)* ) is a predictor of the dependent variable gross language score. The equation that describes this relationship is (raw language score) = 26.644 + 0.318 (resources that promote proximal processes).

**Table 5:** Multiple regression analysis for predicting dependent variables raw scores of orientation and language

Model		Coefficients not Standardized		Coefficients Standardized	t	p-value
		B	Error	Beta		
Gross score of orientation	(Constant)	2.497	0.378		6.613	0.000*
	Predictable activities that signal some degree of stability in family life	0.036	0.018	0.189	2.017	0.046*
Gross score of language	(Constant)	26.644	4.479		5.948	0.000*
	Resourced that promote proximal processes	0.318	0.144	0.206	2.204	0.030*

Multiple linear regression; backwards method

* = Statistically significant value (p < 0.05)

**Caption:** B = Coefficients not Standardized; Error = Standard error; Beta = Coefficients Standardized; t = t-test; p-value = test of significance

The final models of the multivariate regressions showed that each point in the activities that signal some degree of stability in family leads to an increase of 0.189 in the gross score of orientation and that the increase of one point in the resources that promote proximal processes reflects the increase of 0.206 points in the raw language score.

There was no predictive model of multiple linear regression by the backward method for the dependent variables gross score of performance of the neuropsychological functions of attention, perception, memory, visuospatial skills, arithmetic skills, verbal fluency and inhibitory control in the analyzed children. The independent variables mother's education, socioeconomic level, resources that promote proximal processes, parenting practices that promote family-school bonding and predictable activities that signal some degree of stability in family were not predictive in these models.

## DISCUSSION

During the reopening of schools after more than one year of closures and the changes brought about during the COVID-19 pandemic, the following questions were asked: “Would the neuropsychological functions of children, fundamental for good school performance, be impaired when returning to school? Would we find any influence of contextual factors of these skills?”. After the analyses we noted a high prevalence of children who showed alertness or deficits in the functions of orientation, memory, attention, language, visuospatial skills, arithmetic skills and verbal fluency. Still, we found influence of contextual factors, such as predictable activities that signal some degree of stability in family and resources that promote proximal processes in children's performance in some neuropsychological functions.

The results obtained from the battery of neuropsychological tests applied in our study indicated a high number of children in the literacy phase who showed alertness or deficits in neuropsychological skills (Table 1). Since the participants of this research are in the first and second years of basic education, some reflections on the results initial literacy process need to be considered.

The results presented (Table 1) show that 58% of the children had a classification suggestive of alert or deficit in the orientation test, which assesses personal orientation, temporal orientation and spatial orientation. A study realized in 2020 with 408 Greek schoolchildren concluded that self-representation, directly related to self-orientation, is a predictor of academic performance^(15)^.

Regarding memory, we found that 62.4% of the children were in an alert or deficit situation (Table 1). Souza and Sisto^(16)^ report that children with impaired information retention tend to present greater difficulties in the organization, manipulation, storage and evocation of previously taught knowledge, impacting on the consolidation of learning. In that study, the authors performed the memory assessment of 80 Brazilian children in the second and third grades of elementary school and showed a positive relationship between memory level and writing performance^(16)^.

Another key skill for schoolchildren is attention^(7)^. Even though our results showed a predominance of children without alterations for this ability (Table 1), we observed a high number of children who presented a score suggestive of alert or deficit (41.88%). Authors found correlations between the performance of attention and executive functions with the reading, writing and calculation tests of Brazilian children who attended the 2nd to 5th grade of elementary school and suggest that these skills can predict the development of school skills^(17)^.

On visuospatial skills, Table 1 shows that 66.67% of children in the initial years of schooling showed alert or deficit. The visuoconstructive abilities result from the association between the fine motor skills and the visuospatial abilities, being linked to the planning and mental organization, as well as the motor execution and perception of the object^(18)^. A study^(18)^, conducted with Brazilian students who attended elementary school, evidenced an association between the visuoconstructive skills of school-age children with the previous neuropsychomotor development and pointed out that the adequate development of these skills help in the acquisition of more complex cognitive competencies in the course of the development and learning of children.

Regarding the executive skills, we found 48.72% of children with alertness or deficits for verbal fluency and 29.91% for inhibitory control. Studies with Brazilian students with and without learning difficulties show that children with deficits in executive functions tend to complain of learning difficulties^(19,20)^, emphasizing the importance of the results of this research.

For language assessments (Table 1), involving questions of phonological awareness, pragmatic aspects, reading and writing, more than half of the children presented alertness or deficit, and almost 40% of the sample was classified as having a significant severity deficit. For the arithmetic skills, we observed alterations in 69.23% of the children, in which 23.08% had a performance suggestive of moderate to severe deficit.

Studies show that early learning problems do not disappear completely without a targeted intervention^(21)^. As the performance in the final school years is a consequence of what was previously acquired, we can assume that a deficient literacy can cause difficulties during the course of academic life. In this context, immediate actions and public policies to improve the Brazilian educational scenario assume an urgent nature. Thus, cognitive and linguistic factors accessible to intervention should be sought to promote students' literacy, mitigate inequalities and contribute to change in the context of teaching and learning in the country^(22)^.

Regarding contextual factors, we performed a multiple linear regression in order to analyze whether the independent variables of maternal schooling, family environment and socioeconomic classification are able to predict the performance of children in neuropsychological skills. We identified predictable activities that signal some degree of stability in family life and were able to positively predict student performance in guidance tasks (Table 5), which allowed us to infer that the presence of routines, meetings and regular family activities increase personal and temporal notions of children's spaces.

The routine helps in building the sense of predictability and controllability^(23)^. A research^(24)^ conducted with 100 Brazilian children who attended from the 2nd to the 4th year of elementary school and evidenced the association between home routine and opportunities for family interactions with the evolution of students in school learning, which reinforces the importance of routines, meetings and regular activities in the family for the personal and temporo-spatial notions of children, Influential factors in the learning process.

Furthermore, the multiple linear regression analysis showed that the features that promote proximal processes were significant predictors of language performance (Table 5). These resources include stimulating experiences and appropriate use of free time, interaction with parents, availability of physical resources such as toys, materials, books, newspapers and magazines and access to programmed learning activities^(11)^.

The other dependent and independent variables of our study showed results that diverged from those previously evidenced in both the multivariate and univariate analyses. First, considering the variables evaluated by the RAF (Table 3), we observe that the resources that promote proximal processes and the predictable activities that signal some degree of stability in family life were not able to predict the performance of the attention functions, perception, memory, arithmetic skills, visuoconstructive skills and executive functions. Furthermore, it was found that the family-school relationship was not a predictor of any of the neuropsychological skills evaluated.

The literature points out the importance of the organization of the routine, of the family school relationship, of the interaction between the parent-child dyad, of the accomplishment of recreational and educational tasks, of the creation of an environment with adequate stimuli for learning and of the construction of a welcoming domestic atmosphere for the school and cognitive development of the children^(11,25,26)^. Our hypothesis for the divergent findings found in this study is mainly linked to sample characteristics and the fact that studies with results that disagree with ours do not aim at the analysis of neuropsychological functions^(25-28)^.

In the analysis of socioeconomic classification, children from families with estimates of household income below one minimum monthly wage (class DE) showed a poorer performance in the executive function of inhibitory control. Other neuropsychological functions are not associated with socioeconomic classification. Researchers who found results similar to ours raised the hypothesis that the results are attributed to the characteristics of the sample, since this finding is not predominant in studies^(29,30)^. The sample in this research was from a single public school in the Federal District, not covering socioeconomic differences of other groups, such as a greater variability of socioeconomic classes and types of school (public and private). Additionally the sample was predominantly concentrated in C1 and C2 classes and only with 22% of the families being within DE and 3.42% in B2, a factor that may have influenced our findings.

Corroborating this hypothesis, a study developed by Piccolo et al^. (29)^ concluded that socioeconomic status (SES) had an impact on IQ, verbal memory, working memory, oral and written language, and executive functions. The study considered greater variability of socioeconomic classes and different types of schools in their analyses. It may well be possible that the group factors which were not considered in our study may well be important for showing the influence of SES on other neuropsychological functions. This differences does not effect the relevance of our findings, on the contrary it demonstrates the need for further studies that consider other sample and contextual variables of analysis.

Finally, in this research there was no relationship between maternal schooling and children's performance in neuropsychological functions. Currently, the literature has discussed the relationship between maternal schooling and performance in language, executive functions and school learning, since the higher level of parental education can influence the frequency, quantity and quality of stimuli^(10,27,29).^ The hypothesis advanced for our results is that they may have been influenced by the homogeneity and convenience of our sample.

### Study limitations

Collection was carried out in only one public school in the Federal District, therefore we suggest caution in generalization of the findings and to interpret them carefully. Our research emphasizes the lack of studies that approach the subject and propose objectives here. We did not find any other study that covered samples from the Federal District-Brazil or that considered any other locations in Brazil.

Further studies are required which address other socioeconomic and contextual variables in the performance of students' neuropsychological skills, as well as research to assess the prevalence of changes in these skills in other populations, considering different ages, grades and schools.

## CONCLUSION

We observed a high prevalence of children in the literacy phase who showed alertness or deficits in the functions of orientation, memory, attention, language, visuospatial skills, arithmetic skills and verbal fluency. We found the influence of contextual factors, such as predictable activities that signal some degree of stability in family are predictors of children's performance in the orientation skill, and resources that promote proximal processes significantly reflect on language performance.

Children from families with an estimated family income of less than one minimum wage (class DE) have worse performance in the executive inhibitory control function. Other neuropsychological functions are not associated with socioeconomic classification. The variables maternal schooling, resources that promote proximal processes, predictable activities that signal some degree of stability in family life, and parenting practices that promote family-school are not related to neuropsychological skills.
