# ERRATUM: The neuropsychological functions of schoolchildren after the reopening of brazilian schools during the Covid-19 pandemic

**DOI:** 10.1590/2317-1782/20242023013en

**Published:** 2024-11-11

Due to an honest mistake by the authors, the article “The neuropsychological functions of schoolchildren after the reopening of brazilian schools during the Covid-19 pandemic” (DOI https://doi.org/10.1590/2317-1782/20232022334en), present in CoDAS 2024;36(2):e20220334, was published with the incorrect approval number of the Research Ethics Committee.

On page 2, where the text reads:

“The present study was approved by the Research Ethics Committee number. 2,499,005.”

It should read:

“The present study was approved by the Research Ethics Committee number. 4,638,542.”

The authors apologize for the error.

