# Supplementary material for: The neuropsychological functions of schoolchildren after the reopening of brazilian schools during the Covid-19 pandemic
Source: Codas. 2023 Nov 10;36(2):e20220334. doi: 10.1590/2317-1782/20232022334en (PMC10715631; doi:10.1590/2317-1782/20232022334en)
Supplement: Translation

# Translation: Funções neuropsicológicas de escolares na reabertura das escolas brasileiras na pandemia da Covid-19

**DOI:** 10.1590/2317-1782/20232022334pt

**Authors:** Maria Rebeca de Carvalho Porto Ribeiro, Letícia Corrêa Celeste, Vanessa de Oliveira Martins Reis

**Affiliations:** 1 Programa de Pós-graduação em Ciências da Reabilitação - PPGCR, Faculdade de Ceilândia - FCE, Universidade de Brasília - UnB - Brasília (DF), Brasil.; 2 Faculdade de Ceilândia - FCE, Universidade de Brasília - UnB - Brasília (DF), Brasil.

**Keywords:** Isolamento Social, Retorno das Aulas, Crianças, Ensino Fundamental, Cognição, Ambiente Familiar

## Abstract

**Objetivo:**

O objetivo desse estudo foi analisar as funções neuropsicológicas de estudantes de uma escola pública do Distrito Federal (Brasil), matriculadas 1º e 2º ano de Ensino Fundamental na reabertura das escolas públicas na pandemia da COVID-19 e a influência dos fatores familiares e contextuais sobre o desempenho dessas habilidades.

**Métodos:**

Participaram 117 estudantes, bem como seus responsáveis. As crianças foram avaliadas presencialmente por meio do Instrumento de Avaliação Neuropsicológica Breve Infantil (NEUPSILIN-Inf). Os pais/responsáveis responderam remotamente ao Inventário de Recursos do Ambiente Familiar e as questões para classificação socioeconômica e de escolaridade materna.

**Resultado:**

Os dados apontam alta prevalência de crianças em fase de alfabetização que apresentam alerta ou déficit das funções de orientação, memória, linguagem, habilidades visuoespaciais, habilidades aritméticas e fluência verbal. Ainda, as atividades previsíveis que sinalizam algum grau de estabilidade na vida familiar são preditoras do desempenho das crianças na habilidade de orientação e os recursos que promovem processos proximais refletem significativamente no desempenho em linguagem. Os resultados sugerem que crianças inseridas em família com estimativas da renda domiciliar abaixo de um salário mínimo apresentam piores desempenhos do controle inibitório.

**Conclusão:**

Foram apresentados e discutidos os impactos das alterações das habilidades neuropsicológicas para o aprendizado das crianças, destacando a necessidade de intervenções imediatas e direcionadas para essas funções. Foram considerados os fatores contextuais que apresentaram influência sobre o desempenho das habilidades neuropsicológicas.

## INTRODUÇÃO

O surgimento da COVID-19 se tornou um desafio a nível mundial. Em março de 2020, a Organização Mundial da Saúde (OMS) declarou pandemia global, sugerindo que medidas imediatas fossem tomadas a fim de atenuar a transmissão do vírus. Nesse contexto, os governos de diversos países utilizaram a quarentena e o distanciamento social como estratégias de combate a COVID-19^(1)^. No Brasil, a autonomia administrativa dos Municípios e Estados permitiu que essas ações fossem implementadas e como consequência as escolas, os estabelecimentos e as empresas foram fechados por um longo período, trazendo mudanças à sociedade e à vida dos indivíduos^(1)^.

Estudos anteriores realizados após pandemias, epidemias e desastres naturais indicaram consequências na economia, na saúde e em diferentes âmbitos da sociedade durante o ocorrido, com impactos que perpetuaram em outras gerações^(2)^. Porém, as perdas educacionais, os impactos no aprendizado e no ambiente familiar após essas crises, bem como as consequências a longo prazo, ainda não foram amplamente rastreados^(2)^.

Alguns estudos levantam a reflexão quanto aos resquícios negativos da pandemia da COVID-19 e das medidas de isolamento no ensino e aprendizado, no qual pesquisadores e organizações projetam que o fechamento das escolas irá trazer prejuízo para as crianças^(2-5)^, com redução da capacidade de leitura^(5)^ e desaceleração dos ganhos de aprendizagem, principalmente para alunos do Ensino Fundamental^(3)^.

Os primeiros anos do Ensino Fundamental são essenciais para a alfabetização das crianças. Nesse momento, busca-se que o estudante compreenda o que lê e escreve e desenvolva as competências necessárias para continuar esse processo de forma autônoma^(6)^. O aprendizado é impactado pela integridade de habilidades neuropsicológicas, que envolvem questões cognitivas, atencionais, linguísticas e de memória^(7)^.

Estudos recentes demonstram relação entre o desempenho da leitura e escrita com aspectos linguístico-cognitivos e sugerem que crianças com melhores desempenhos em linguagem e cognição tendem a apresentar melhores índices de leitura e escrita^(8-10)^. Uma pesquisa^(9)^ realizada em 2018 com escolares brasileiros mostrou que crianças com dificuldade de aprendizagem apresentam déficits na memória e em habilidades metacognitivas. Outro estudo publicado em 2020^(10)^, também com estudantes brasileiros, evidenciou que algumas variáveis cognitivas, como consciência fonológica e a nomeação automática, são preditores de leitura.

Sendo o indivíduo influenciado pelo meio em que está inserido, pode-se imaginar as repercussões do cenário pandêmico e do fechamento das escolas para a aquisição de habilidades neuropsicológicas, fundamentais para o processo de alfabetização e para o bom desempenho escolar.

Dadas as preocupações da comunidade acadêmica quanto aos possíveis impactos negativos do isolamento para as crianças e diante das retomadas graduais das aulas presenciais, o objetivo do presente estudo foi verificar e analisar a prevalência de crianças, no início da alfabetização, com déficits nas funções neuropsicológicas no momento da reabertura das escolas públicas na pandemia da COVID-19, bem como a influência dos fatores familiares e contextuais.

## MÉTODO

Participaram do estudo 117 crianças, de ambos os sexos, sendo 59 do 1º ano e 58 do 2º ano do Ensino Fundamental de uma escola pública do Distrito Federal (Brasil), bem como seus pais/responsáveis. Foi considerado como critério de inclusão estar devidamente matriculada na escola na qual o estudo foi desenvolvido, em turma regular ou inclusiva. Como critério de exclusão considerou-se as crianças com dificuldades visuais não corrigidas e deficiências intelectuais e/ou auditivas, relatados pelos responsáveis ou pela escola, e a resposta incompleta dos protocolos.

A fim de alcançar os objetivos deste estudo, os pais ou responsáveis responderam remotamente a uma entrevista contendo questões de dados gerais e escolaridade materna, além do Inventário de Recursos do Ambiente Familiar (RAF) e o critério de Classificação Econômica Brasil (CCEB). A coleta de dados dos estudantes foi realizada presencialmente por meio do Instrumento de Avaliação Neuropsicológica Breve Infantil (NEUPSILIN- Inf).

O Inventário de Recursos do Ambiente Familiar (RAF) é composto por dez questões de itens abertos e de múltipla escolha, que tem por objetivo avaliar a rotina da criança e seu ambiente familiar^(11)^. O questionário é agrupado em três grandes domínios: os recursos que promovem processos proximais, atividades previsíveis que sinalizam algum grau de estabilidade na vida familiar e práticas parentais que promovem a ligação família-escola^(11)^.

O Critério de Classificação Econômica Brasil (CCEB) tem o objetivo de avaliar o poder de compra de grupos de consumidores, dividindo-os em classes econômicas. É composto por questões que resultam em uma pontuação final que classifica o respondente nas classes A, B1, B2, C1, C2 e D-E^(12)^.

O Instrumento de Avaliação Neuropsicológica Breve Infantil (NEUPSILIN- Inf) é um protocolo de curta duração, destinado a avaliar oito funções neuropsicológicas de crianças com idade entre seis e 12 anos. É composto por 26 subtestes que envolvem as habilidades de orientação; atenção; percepção visual e de emoções; memória verbal e visual, subdividida em memória de trabalho, episódica e semântica; habilidades aritméticas; linguagem, composta pela linguagem oral, leitura e escrita; habilidades visuoconstrutivas; e funções executivas, que incluem controle inibitório e fluência verbal^(13)^.

Os participantes foram recrutados via ligação telefônica, na qual os pais ou responsáveis receberam informações sobre a pesquisa e tiveram suas dúvidas esclarecidas. Para os que concordaram em participar, foi agendada a aplicação da entrevista, com questões de dados gerais e da escolaridade materna, e dos questionários (CCEB e RAF).

Após a entrevista, foi agendado o horário para a criança ser avaliada com o NEUPSILIN-Inf na própria escola. Com a flexibilização das medidas de fechamento das escolas após 15 meses de isolamento social e a criação de um protocolo de segurança bem estabelecido foi possível iniciar a coleta presencial com as crianças em Junho/2021. O protocolo foi aplicado seguindo comandos e estímulos padronizados no livro de aplicação.

O presente estudo foi aprovado pelo Comitê de Ética em Pesquisa da instituição sob parecer nº 2.499.005. Todos os participantes estavam cientes dos procedimentos e objetivos do estudo e assinaram o termo de consentimento livre e esclarecido (TCLE) destinado aos responsáveis e alunos e termo de assentimento livre e esclarecido (TALE) destinado aos alunos.

Para a análise dos dados, os subitens do NEUPSILIN-Inf foram pontuados quanto ao escore bruto e ao escore Z de acordo com os critérios do teste^(13)^. Cada escore Z foi classificado em: sugestivo de alerta para déficit neuropsicológico (escore z entre -1,0 e -1,5); sugestivo de déficit (escore z menor ou igual a -1,5); sugestivo de alerta para déficit moderado a severo (escore z entre -1,6 e -2,0); e sugestivo de déficit de gravidade importante (escore z menor ou igual a -2,0).

Frente a ausência de dados normativos para a população do Distrito Federal para a análise do NEUPSILIN-Inf, foram considerados os dados de referência dos estudantes do Rio Grande do Sul, levando em consideração a idade (seis a oito anos) e o tipo de escola (pública). Esta escolha levou em conta o Índice de Desenvolvimento da Educação Básica (IDEB) para os anos iniciais de 2019^(14)^. O Distrito Federal apresentou pontuação 6,1 no IDEB para os anos iniciais; Rio Grande do Sul 5,8; e São Paulo 6,5.

As variáveis independentes contextuais consideradas para o estudo foram: escolaridade materna, ambiente familiar e classificação socioeconômica. Em relação à escolaridade materna, consideramos o agrupamento da realização parcial ou completa do Ensino Fundamental, Ensino Médio e Ensino Superior/Pós-Graduação.

Para a análise do ambiente familiar, utilizamos a pontuação obtida nos três grandes domínios do RAF, agrupando as questões de acordo com a divisão proposta pelo protocolo, a saber: os recursos que promovem processos proximais (questões 1,2,3,4,5,6,7), atividades previsíveis que sinalizam algum grau de estabilidade na vida familiar (questões 9, 10) e práticas parentais que promovem a ligação família-escola (questão 8). Por fim, para a análise considerando as classificações socioeconômicas, realizamos o agrupamento das classes B1, C1 e C2, que apresentavam previsão de renda domiciliar acima de um salário-mínimo, e o agrupamento das classes D e E, que apresentavam previsão de renda domiciliar abaixo de um salário-mínimo.

Para análise dos dados foi utilizado o software SPSS 25.0, considerando um nível de significância de 5% para as análises inferenciais. Na análise descritiva das variáveis quantitativas foram calculadas as medidas de tendência central, variabilidade e posição.

A correlação entre as variáveis quantitativas não-normais foi realizada com o Teste de Correlação de Spearman. A análise inferencial de comparação das variáveis quantitativas não-normais entre dois grupos independentes foi realizada com o Teste de Mann-Whitney. A análise inferencial de comparação das variáveis quantitativas não-normais em função de múltiplos grupos independentes foi realizada com o Teste de Kruskal-Wallis.

Realizaram-se análises de regressão linear múltipla pelo método backward para prever as variáveis dependentes escore bruto de desempenho das funções neuropsicológicas de orientação, atenção, percepção, memória, habilidades visuoespaciais, habilidades aritméticas, fluência verbal, linguagem e controle inibitório a partir das variáveis independentes escolaridade da mãe, critério econômico do Brasil, recursos que promovem processos proximais, relação família-escola e atividades que sinalizam algum grau de estabilidade familiar. As variáveis com múltiplas categorias foram transformadas em binárias pelo método dummy.

## RESULTADOS

Participaram do estudo 117 crianças em fase de alfabetização, com idades entre seis e oito anos, média de seis anos e nove meses. Os estudantes tiveram frequência equilibrada de escolaridade no primeiro (n=59; 50,43%) e segundo (n=58; 49,57%) anos do Ensino Fundamental.

Quanto à classificação econômica das famílias, a categoria mais frequente foi a C2 (n=60; 51,28%), seguida por C1 (n=27;23,08%), por DE (n=26;22,22%) e por B2 (n=4;3,42%). Nossa amostra não apresentou participantes classificados em A e B1.

A escolaridade materna foi predominantemente de Ensino Médio (n=63; 53,80%), contando também com mães que apresentavam Ensino Fundamental (n=12; 10,70%) e Ensino Superior ou Pós-Graduação (n=37; 33,00%).

A Tabela 1 apresenta a prevalência de alerta e déficit em cada uma das funções neuropsicológicas avaliadas pelo NEUPSILIN-Inf. Observa-se um maior número de estudantes com classificação sugestiva de alerta ou déficit neuropsicológico para os escores Z de orientação, memória, linguagem, habilidades visuoespaciais, aritmética e fluência verbal.

**Tabela 1:** Prevalência de alerta e déficit em funções neuropsicológicas em crianças do 1º e 2º ano de uma escola pública do Distrito Federal- Brasil a partir do NEUPSILIN-Inf

**Variável e categorias**	**n**	**Por categoria (%)**	**Total (%)**
**Orientação**			
Alerta para déficit neuropsicológico	54	46,15	58,12
Déficit de gravidade importante	14	11,97	
**Atenção**			
Alerta para déficit neuropsicológico	13	11,11	41,88
Déficit neuropsicológico	5	4,27	
Déficit moderado a severo	6	5,13	
Déficit de gravidade importante	25	21,37	
**Percepção**			
Déficit moderado a severo	12	10,26	17,10
Déficit de gravidade importante	8	6,84	
**Memória**			
Alerta para déficit neuropsicológico	16	13,68	62,40
Déficit neuropsicológico	5	4,27	
Déficit moderado a severo	10	8,55	
Déficit de gravidade importante	42	35,9	
**Linguagem**			
Alerta para déficit neuropsicológico	14	11,97	62,24
Déficit neuropsicológico	6	5,13	
Déficit moderado a severo	15	12,82	
Déficit de gravidade importante	46	39,32	
**Habilidades visuoespaciais**			
Alerta para déficit neuropsicológico	8	6,84	66,67
Déficit moderado a severo	11	9,4	
Déficit de gravidade importante	59	50,43	
**Aritmética**			
Alerta para déficit neuropsicológico	39	33,33	69,23
Déficit neuropsicológico	5	4,27	
Déficit moderado a severo	27	23,08	
Déficit de gravidade importante	10	8,55	
**Fluência verbal**			
Alerta para déficit neuropsicológico	18	15,38	51,28
Déficit neuropsicológico	2	1,71	
Déficit moderado a severo	11	9,4	
Déficit de gravidade importante	29	24,79	
**Controle inibitório**			
Alerta para déficit neuropsicológico	7	5,98	29,91
Déficit neuropsicológico	2	1,71	
Déficit moderado a severo	3	2,56	
Déficit de gravidade importante	23	19,66	

**Legenda:** n=frequência absoluta; %=frequência relativa

As Tabelas 2 e 3 apresentam os resultados da análise de associação entre os domínios do RAF e o desempenho nas funções neuropsicológicas. Não houve correlação entre escores brutos de desempenho nas funções neuropsicológicas e os recursos que promovem processos proximais, as práticas parentais que promovem a ligação família-escola e as atividades previsíveis que sinalizam algum grau de estabilidade na vida familiar na amostra estudada (Tabela 3).

**Tabela 2:** Análise descritiva das variáveis recursos que promovem processos proximais, as práticas parentais que promovem a ligação família-escola e as atividades previsíveis que sinalizam algum grau de estabilidade na vida familiar e das habilidades neuropsicológicas

**Variável**	**Média**	**DP**	**Mínimo**	**Máximo**	**1Q**	**Mediana**	**3Q**
**RAF**							
Recursos que promovem processos proximais	29,23	10,87	5	54	20	31	38
Relação família-escola	12,24	2,46	6	18	11,5	12	13
Atividades que sinalizam algum grau de estabilidade familiar	20,4	5,53	1	28	17,5	21	24
**NEUPSILIN-Inf**							
Orientação	3,21	1,06	0	6	3	3	4
Atenção	44,06	7,98	19	59	38,5	45	51
Percepção	5,82	0,41	4	6	6	6	6
Memória	40,59	17,12	5	85	28,5	40	51,5
Linguagem	35,21	17,08	0	65	20	31	52
Habilidades visuoespaciais	15,58	4,06	0	23	14	16	18
Aritmética	3,56	3,44	0	18	1	2	6
Fluência verbal	10,84	5,46	0	26	7	10	14
Controle inibitório	40,73	16,94	0	59	39	46	52

**Legenda:** DP=desvio padrão; 1Q=primeiro quartil; 3Q=terceiro quartil

**Tabela 3:** Correlação entre as variáveis escore bruto de desempenho nas funções neuropsicológicas e os domínios do RAF

	**Recursos que promovem processos proximais**	
	r	**p-valor**
Orientação	0,075	0,422
Atenção	0,015	0,877
Percepção	-0,005	0,954
Memória	0,116	0,213
Linguagem	0,174	0,061
Habilidades visuoespaciais	-0,036	0,704
Aritméticas	0,151	0,103
Fluência verbal	-0,075	0,422
Controle inibitório	0,135	0,146
	**Relação família-escola**	
	**r**	**p-valor**
Orientação	-0,080	0,392
Atenção	-0,072	0,438
Percepção	-0,119	0,200
Memória	-0,179	0,053
Linguagem	-0,030	0,752
Habilidades visuoespaciais	-0,135	0,147
Aritméticas	-0,099	0,288
Fluência verbal	-0,057	0,541
Controle inibitório	-0,170	0,067
	**Atividades que sinalizam algum grau de estabilidade familiar**	
	**r**	**p-valor**
Orientação	0,156	0,094
Atenção	0,052	0,574
Percepção	0,050	0,594
Memória	0,081	0,386
Linguagem	0,153	0,101
Habilidades visuoespaciais	0,052	0,580
Aritméticas	0,099	0,289
Fluência verbal	0,101	0,281
Controle inibitório	0,105	0,261

Teste de Correlação de Spearman

**Legenda:** r=coeficiente de correlação; p-valor = teste de significância

Na análise socioeconômica, as crianças das classes B2/C1/C2 apresentaram escores significativamente maiores na função neuropsicológica de controle inibitório que as crianças das classes D/E (p=0,020) (Tabela 4). Não foi observada diferença na comparação dos escores brutos de desempenho das habilidades neuropsicológicas em função da escolaridade das mães das crianças.

**Tabela 4:** Análise inferencial de comparação das variáveis escores brutos de desempenho nas funções neuropsicológicas em função das variáveis nível socioeconômico e escolaridade materna

**Funções neuropsicológicas**	**Nível socioeconômico**	**Média**	**DP**	**Mínimo**	**Máximo**	**1Q**	**Mediana**	**3Q**	**p-valor**
Orientação	B2/C1/C2	3,18	0,98	0	6	3	3	4	0,611
	DE	3,31	1,32	1	6	2	3	4	
Atenção	B2/C1/C2	43,67	7,96	19	59	38	45	50	0,372
	DE	45,42	8,09	28	59	38,75	46	51,25	
Percepção	B2/C1/C2	5,82	0,41	4	6	6	6	6	0,763
	DE	5,81	0,4	5	6	6	6	6	
Memória	B2/C1/C2	41,18	15,59	5	85	30	42	51	0,364
	DE	38,54	21,89	11	74	20,5	31	63,25	
Linguagem	B2/C1/C2	35,23	16,42	0	65	21	31	50	0,665
	DE	35,15	19,56	10	64	17	30,5	55,25	
Habilidades visuoespaciais	B2/C1/C2	15,68	3,86	0	23	14	16	18	0,775
	DE	15,23	4,77	0	22	12	15,5	18,25	
Aritmética	B2/C1/C2	3,48	3,26	0	18	1	2	5,5	0,678
	DE	3,85	4,07	0	11	1	1	9	
Fluência verbal	B2/C1/C2	10,71	5,34	0	25	7	10	14	0,753
	DE	11,27	5,97	0	26	6	10,5	15,25	
Controle inibitório	B2/C1/C2	42,15	16,13	0	59	41	47	52	0,02*
	DE	35,73	19,02	0	53	31,25	43,5	49,25	
							

Teste de Kruskal-Wallis

* = Valor estatisticamente significativo (p < 0,05)

**Legenda:** DP=desvio padrão; 1Q=primeiro quartil; 3Q=terceiro quartil

Foi realizada a regressão linear múltipla para verificar se as variáveis independentes são capazes de prever as variáveis dependente escore bruto de orientação e escore bruto de linguagem (Tabela 5). A análise resultou em um modelo estatisticamente significativo, tanto para orientação *[F(1, 110) = 4,069; p=0,046; R2 = 0,036],* quanto para linguagem *[F(1, 110) = 4,859; p=0,030; R2 = 0,042].* A variável independente de atividades previsíveis que sinalizam algum grau de estabilidade na vida familiar *(β = 0,189; t = 2,017; p = 0,046)* é previsora da variável dependente escore bruto de orientação. A equação que descreve essa relação é (escore bruto de orientação) = 2,497 + 0,036 (atividades que sinalizam algum grau de estabilidade familiar)*.* A variável independente recursos que promovem processos proximais *(β = 0,206; t = 2,204; p = 0,030)* é previsora da variável dependente escore bruto de linguagem. A equação que descreve essa relação é *(escore bruto de linguagem) = 26,644 + 0,318 (recursos que promovem processos proximais).*

**Tabela 5:** Análise de regressão múltipla para previsão das variáveis dependentes escores brutos de orientação e de linguagem

Modelo		Coeficientes não padronizados		Coeficientes padronizados	t	p-valor
		B	Erro	Beta		
Escore bruto de orientação	(Constante)	2,497	0,378		6,613	0,000*
	Atividades que sinalizam algum grau de estabilidade familiar	0,036	0,018	0,189	2,017	0,046*
Escore bruto de linguagem	(Constante)	26,644	4,479		5,948	0,000*
	Recursos que promovem processos proximais	0,318	0,144	0,206	2,204	0,030*

Regressão linear múltipla; método backward

* = Valor estatisticamente significativo (p < 0.05)

**Legenda:** B = Coeficientes não Padronizados; Erro = Erro padrão; Beta = Coeficientes Padronizados; t = teste t; p-valor = teste de significância

Dessa forma, os modelos finais das regressões multivariada, apontaram que cada um ponto nas atividades previsíveis que sinalizam algum grau de estabilidade na vida familiar leva a um aumento de 0,189 no escore bruto de orientação e que o aumento de um ponto nos recursos que promovem processos proximais reflete o aumento de 0,206 pontos no escore bruto de linguagem.

Não houve modelo preditivo de regressão linear múltipla pelo método backward para as variáveis dependentes escore bruto de desempenho das funções neuropsicológicas de atenção, percepção, memória, habilidades visuoespaciais, habilidades aritméticas, fluência verbal e controle inibitório nas crianças analisadas. As variáveis independentes escolaridade da mãe, nível socioeconômico, recursos que promovem processos proximais, as práticas parentais que promovem a ligação família-escola e as atividades previsíveis que sinalizam algum grau de estabilidade na vida familiar não foram preditivas nesses modelos.

## DISCUSSÃO

Durante a reabertura das escolas após mais de um ano fechadas e as mudanças enfrentadas durante a pandemia da COVID-19, foram levantadas as seguintes questões: “As funções neuropsicológicas das crianças, fundamentais para o bom desempenho escolar, estariam prejudicadas no retorno às aulas? Encontraríamos influência dos fatores contextuais nessas habilidades?”. Após as análises, observamos uma alta prevalência de crianças que apresentaram alerta ou déficit nas funções de orientação, memória, atenção, linguagem, habilidades visuoespaciais, habilidades aritméticas e fluência verbal. Encontramos também influência dos fatores contextuais, como atividades previsíveis que sinalizam algum grau de estabilidade na vida familiar e os recursos que promovem processos proximais, no desempenho das crianças em algumas funções neuropsicológicas.

Os resultados obtidos na bateria de testes neuropsicológicos aplicada em nosso estudo apontaram um número alto de crianças na fase de alfabetização que apresentam alerta ou déficit em habilidades neuropsicológicas (Tabela 1). Visto que os participantes desta pesquisa se encontram no primeiro e segundo ano do Ensino Fundamental, algumas reflexões sobre os achados e o processo de aprendizado inicial da alfabetização precisam ser consideradas.

Os resultados apresentados (Tabela 1) mostram que 58% das crianças apresentaram classificação sugestiva de alerta ou déficit na prova de orientação, que avalia a orientação pessoal, temporal e espacial. Uma pesquisa realizada em 2020 com 408 escolares gregos concluiu que a autorrepresentação, diretamente relacionada à orientação de si mesmo, é um preditor de performance acadêmica^(15)^.

Para memória, encontramos que 62,4% das crianças estavam em situação de alerta ou déficit (Tabela 1). Souza e Sisto^(16)^ relatam que crianças com prejuízo na retenção da informação tendem a apresentar maiores dificuldades na organização, na manipulação, no armazenamento e na evocação de conhecimentos previamente ensinados, impactando na consolidação do aprendizado. Nesse estudo, os autores realizaram a avaliação de memória de 80 crianças brasileiras da segunda e terceira séries do Ensino Fundamental e evidenciaram relação positiva entre o nível de memória e o desempenho na escrita^(16)^.

Outra habilidade primordial para os escolares é a atenção^(7)^. Mesmo que os nossos resultados tenham apresentado predominância de crianças sem alterações para essa habilidade (Tabela 1), observamos um número alto de crianças que apresentaram escore sugestivo de alerta ou déficit (41,88%). Autores encontraram correlações entre o desempenho da atenção e das funções executivas com os testes de leitura, escrita e cálculo de crianças brasileiras que cursavam do 2º ao 5º ano do Ensino Fundamental e sugerem que essas habilidades podem prever o desenvolvimento das competências escolares^(17)^.

Para as habilidades visuoespaciais, a tabela 1 aponta que 66,67% das crianças nos anos iniciais de escolaridade apresentaram alerta ou déficit. As habilidades visuoconstrutivas resultam da associação entre as habilidades motoras finas e as habilidades visuoespaciais, estando vinculadas ao planejamento e organização mental, bem como a execução motora e percepção do objeto^(18)^. Um estudo^(18)^, realizado com estudante brasileiros que cursavam o Ensino Fundamental, evidenciou uma associação entre as habilidades visuoconstrutivas de crianças em idade escolar com o desenvolvimento neuropsicomotor prévio e apontou que o adequado desenvolvimento dessas habilidades auxiliam na aquisição de competências cognitivas mais complexas no decorrer do desenvolvimento e aprendizado das crianças.

Por fim, para as habilidades executivas, encontramos 48,72% das crianças com alerta ou déficit para a fluência verbal e 29,91% para o controle inibitório. Estudos com escolares brasileiros com e sem dificuldades de aprendizagem mostraram que crianças com déficit nas funções executivas tendem a apresentar queixas de dificuldade de aprendizagem^(19,20)^, enfatizando a importância dos resultados desta pesquisa.

Para as avaliações de linguagem (Tabela 1), envolvendo questões de consciência fonológica, aspectos pragmáticos, leitura e escrita, mais da metade das crianças apresentaram alerta ou déficit, sendo que quase 40% da amostra estava classificada com déficit de gravidade importante. Para as habilidades aritméticas observamos alteração em 69,23% das crianças, nas quais 23,08% estavam com desempenho sugestivo de déficit moderado a severo.

A literatura aponta que os problemas de aprendizagem iniciais não desaparecem por completo sem que haja uma intervenção direcionada^(21)^. Sendo o desempenho nos anos escolares finais consequência do que foi adquirido anteriormente, podemos pressupor que uma alfabetização deficitária, poderá acarretar dificuldades no decorrer da vida acadêmica. Nesse contexto, ações e políticas públicas imediatas para melhorar o cenário educacional brasileiro assumem caráter de urgência. Dessa forma, deve-se buscar fatores cognitivos e linguísticos acessíveis à intervenção para promover a alfabetização dos alunos, atenuar as desigualdades e contribuir para a mudança no contexto de ensino e aprendizagem no país^(22)^.

Em relação aos fatores contextuais, realizamos uma regressão linear múltipla a fim de analisar se as variáveis independentes de escolaridade materna, ambiente familiar e classificação socioeconômica são capazes de prever o desempenho das crianças nas habilidades neuropsicológicas. Identificamos que as atividades que sinalizam algum grau de estabilidade familiar conseguiram prever positivamente o desempenho dos estudantes nas tarefas de orientação (Tabela 5), o que nos permite inferir que a presença de rotinas, reuniões e atividades regulares em família aumentam as noções pessoais e têmporo-espaciais das crianças.

A rotina auxilia na construção do senso de previsibilidade e controlabilidade^(23)^. Uma pesquisa^(24)^ realizada com 100 crianças brasileiras que cursavam do 2º ao 4º ano do ensino fundamental e evidenciou a associação entre rotina do lar e oportunidades de interações familiares com a evolução dos estudantes na aprendizagem escolar, o que reforça a importância das rotinas, reuniões e atividades regulares em família para as noções pessoais e têmporo-espaciais das crianças, fatores influentes no processo de aprendizagem.

A análise de regressão linear múltipla também apontou que os recursos que promovem processos proximais foram previsores significativos do desempenho em linguagem (Tabela 5). Esses recursos incluem experiências estimuladoras e uso adequado do tempo livre; interação com os pais; disponibilidade de recursos físicos, como brinquedos, materiais, livros, jornais e revistas e acesso a atividades programadas de aprendizagem^(11)^.

As demais variáveis dependentes e independentes do nosso estudo apontaram resultados divergentes aos anteriormente evidenciados tanto na análise multivariada quanto na univariadas. Primeiramente, considerando as variáveis avaliadas pelo RAF (Tabela 3), observamos que os recursos que promovem processos proximais e as atividades que sinalizam algum grau de estabilidade familiar não foram capazes de prever o desempenho nas funções de atenção; percepção; memória; habilidades aritméticas; habilidades visuoconstrutivas; e funções executivas. Além disso, verificou-se que a relação família-escola não foi preditora de nenhuma das habilidades neuropsicológicas avaliadas.

A literatura aponta a importância da organização da rotina, da relação família escola, da interação entre a díade pais-filhos, da realização de tarefas recreativas e educativas, da criação de um ambiente com estímulos adequados para o aprendizado e a da construção de uma atmosfera doméstica acolhedora para o desenvolvimento escolar e cognitivo das crianças^(11,25,26)^. Nossa hipótese para os achados divergentes encontrados neste estudo está ligada principalmente as características amostrais e ao fato das pesquisas com resultados discordantes ao nosso não terem como objetivo a análise das funções neuropsicológicas^(25-28)^.

Na análise da classificação socioeconômica, as crianças inseridas em família com estimativas da renda domiciliar abaixo de um salário-mínimo (classe DE) apresentam piores desempenhos da função executiva de controle inibitório. As demais funções neuropsicológicas não apresentam associação com a classificação socioeconômica. Autores que encontraram resultados semelhantes aos nossos levantam a hipótese de os resultados estarem atribuídos às características da amostra, uma vez que esse achado não é predominante na literatura^(29,30)^. A amostra desta pesquisa foi de uma única escola pública do Distrito Federal, não abrangendo outras diferenças socioeconômicas grupais como uma variabilidade maior de classes socioeconômicas e tipos de escola (público e privada). Demais, a amostra estava predominantemente concentrada na classe C1 e C2, sendo que apenas 22% das famílias estavam dentro de DE e 3,42% em B2, fator que pode ter influenciado os nossos achados.

Corroborando essa hipótese, um estudo desenvolvido por Piccolo *et al.* ^(29)^ concluiu que o status socioeconômico (SES) teve impacto no QI, na memória verbal, na memória de trabalho, na linguagem oral e escrita e nas funções executivas. O estudo considerou uma variabilidade maior de classes socioeconômicas e tipos diferentes de escola em suas análises. Dessa forma, levantamos a possibilidade de fatores grupais não considerados no nosso estudo serem importantes para a manifestação da influência da SES nas demais funções neuropsicológicas. As diferenças expostas não excluem a relevância dos nossos achados, porém atribui visibilidade a necessidade de mais estudos que considerem outras variáveis amostrais e contextuais de análise.

Por fim, nesta pesquisa não foi observado relação da escolaridade materna com o desempenho das crianças nas funções neuropsicológicas. Atualmente, a literatura tem discutido sobre a relação entre a escolaridade materna e o desempenho na linguagem, nas funções executivas e no aprendizado escolar, uma vez que o maior nível de escolaridade parental, pode influenciar a frequência, a quantidade e a qualidade de estímulos^(10,27,29)^. A hipótese aventada para os nossos resultados é que estes podem ter sido influenciados pela característica de homogeneidade e de conveniência da nossa amostra.

### Limitações do estudo

A coleta foi realizada em uma única escola pública do Distrito Federal. Dessa forma, sugerimos cautela na generalização dos achados e uma interpretação cuidadosa deles. Nossa pesquisa enfatiza a carência de estudos que abordem a temática e os objetivos aqui propostos uma vez que não encontramos nenhum estudo que abrangesse amostras do Distrito Federal ou que considerassem outras localidades do Brasil.

Salienta-se que são necessários novos estudos abordando outras variáveis socioeconômicas e contextuais no desempenho das habilidades neuropsicológicas de alunos, bem como pesquisas que avaliem a prevalência das alterações dessas habilidades em outras populações, considerando idades, séries e escolas distintas.

## CONCLUSÃO

Observamos alta prevalência de crianças em fase de alfabetização que apresentam alerta ou déficit das funções de orientação, memória, atenção, linguagem, habilidades visuoespaciais, habilidades aritméticas e fluência verbal. Também evidenciamos que as atividades previsíveis que sinalizam algum grau de estabilidade na vida familiar são preditoras do desempenho das crianças na habilidade de orientação e os recursos que promovem processos proximais refletem significativamente no desempenho em linguagem.

Crianças inseridas em família com estimativas da renda domiciliar abaixo de um salário mínimo (classe DE) apresentam piores desempenhos da função executiva de controle inibitório. As demais funções neuropsicológicas não apresentam associação com a classificação socioeconômica. As variáveis de escolaridade materna, recursos que promovem processos proximais, atividades previsíveis que sinalizam algum grau de estabilidade na vida familiar e práticas parentais que promovem a ligação família-escola não apresentam relação com as habilidades neuropsicológicas.
