# Supplementary material for: ERRATUM: The neuropsychological functions of schoolchildren after the reopening of brazilian schools during the Covid-19 pandemic
Source: Codas. 2024 Nov 11;36(6):e20230013. doi: 10.1590/2317-1782/20242023013en (PMC11610595; doi:10.1590/2317-1782/20242023013en)
Supplement: Translation

# Translation: ERRATA: Funções neuropsicológicas de escolares na reabertura das escolas brasileiras na pandemia da Covid-19

**DOI:** 10.1590/2317-1782/20242023013pt

Devido a erro honesto dos autores, o artigo “Funções neuropsicológicas de escolares na reabertura das escolas brasileiras na pandemia da Covid-19” (DOI https://doi.org/10.1590/2317-1782/20232022334pt), presente em CoDAS 2024;36(2):e20220334, foi publicado com o número de aprovação do Comitê de Ética em Pesquisa incorreto.

Na página 2, onde se lia:

“O presente estudo foi aprovado pelo Comitê de Ética em Pesquisa da instituição sob parecer nº 2.499.005.”

Leia-se:

“O presente estudo foi aprovado pelo Comitê de Ética em Pesquisa da instituição sob parecer nº 4.638.542.”

Os autores pedem desculpas pelo erro.
